# Elevated DUOX2 levels correlate with necrotizing enterocolitis development

**DOI:** 10.3389/fcimb.2025.1689969

**Published:** 2025-11-26

**Authors:** Wenqiang Sun, Xue Liu, Jiahui Huang, Yihui Li, Wei Zhai, Xinyun Jin, Lili Li, Xueping Zhu

**Affiliations:** 1 Department of Neonatology, Children’s Hospital of Soochow University, Suzhou, China; 2 Suzhou Municipal Key Laboratory of Neonatal Major Disease Rescue and Early-Life Prevention of Adult Chronic Diseases, Suzhou, China; 3 School of Basic Medicine, Qingdao University, Qingdao, China; 4 Department of Neonatology, the Affiliated Suzhou Hospital of Nanjing Medical University, Suzhou, China

**Keywords:** necrotizing enterocolitis, dual oxidase 2, immunity, serum, biomarker

## Abstract

**Background:**

Neonatal necrotizing enterocolitis (NEC) is a life-threatening intestinal disease primarily affecting preterm infants. Although the exact pathophysiology remains unclear, immune responses and redox reactions play crucial roles in its development.

**Methods:**

We identified Dual Oxidase 2 (DUOX2) as a significantly upregulated gene in the intestinal tissues of NEC patients. Subsequent analyses included Gene Ontology (GO) enrichment and immune infiltration profiling related to DUOX2. Using a nested case-control design, we then evaluated the clinical relevance of serum DUOX2 protein levels in preterm infants with NEC (gestational age <32 weeks).

**Results:**

DUOX2 was significantly highly expressed in NEC intestinal tissues, with an AUC value of 0.844 (0.599-1.000) predicting the occurrence of NEC. GO enrichment analysis revealed that the differentially expressed genes (DEGs) between the NEC and control groups mainly affect redox reactions, including reactive oxygen species (ROS) and superoxide. Immunoinfiltration analysis revealed a positive correlation between DUOX2 expression and plasma cells (R=0.69, *P*=0.041). Univariate and multivariate analyses identified high levels of serum DUOX2 as a risk factor for developing NEC. Serum DUOX2 levels were negatively correlated with albumin levels and positively correlated with red blood cell distribution width and lactate dehydrogenase levels. The AUC value of serum DUOX2 levels for diagnosing NEC was 0.809 (95% CI: 0.712–0.906), with an optimal cut-off value of 2.483 ng/mL, a sensitivity of 91.30%, and a specificity of 78.26%.

**Conclusion:**

This study demonstrates a significant association between elevated DUOX2 expression and the development of NEC in preterm infants.

## 1 Introduction

Necrotizing enterocolitis (NEC) is a common and severe acute intestinal condition in neonatal intensive care units (NICUs), primarily affecting preterm infants. Currently, there are no specific therapeutic interventions for this disease, and surviving infants often suffer from adverse outcomes (Zozaya et al., 2021; Bell et al., 2022; Itriago et al., 2023; Roberts et al., 2024; Wang et al., 2024). Continued exploration of novel biomarkers is crucial for the screening and identification of NEC. Therefore, in this study, we conducted a preliminary analysis of the transcriptome database of the NEC population and initially identified Dual Oxidase 2 (DUOX2) as a gene with a large fold change that is upregulated in NEC.

DUOX2, a member of the NADPH oxidase family, generates hydrogen peroxide at mucosal surfaces and plays a multifaceted role in intestinal physiology. It contributes to gut microbial homeostasis by producing reactive oxygen species (ROS) that limit microbial overgrowth and maintain spatial organization of the microbiota; at the same time, aberrant DUOX2 activity can disturb redox balance and induce oxidative stress, leading to epithelial barrier dysfunction (Hu et al., 2024). Moreover, DUOX2 is closely linked to inflammatory responses, as its upregulation in conditions such as inflammatory bowel disease enhances ROS-mediated immune activation and cytokine release (Grasberger et al., 2021; Phi Dang et al., 2025). Together, these findings highlight DUOX2 as a central mediator connecting microbial regulation, oxidative stress, and intestinal inflammation.

Subsequently, we measured serum DUOX2 levels in NEC patients with a gestational age (GA) of <32 weeks using Enzyme-Linked Immunosorbent Assay (ELISA), and found that DUOX2 was significantly elevated in the serum of children with NEC. It was identified as an independent risk factor for the development of NEC and was correlated with red blood cell distribution width (RDW), lactate dehydrogenase (LDH), and albumin levels in these children. These findings suggest that elevated DUOX2 levels correlate with NEC development in premature infants, though further studies are needed to evaluate its specificity compared to other neonatal intestinal diseases.

## 2 Materials and methods

### 2.1 Study population

This study was conducted as a nested case-control design. Preterm infants with a gestational age (GA) of <32 weeks who were hospitalized at the Children’s Hospital of Soochow University and Soochow Hospital of Nanjing Medical University within 24 hours after birth between January 1, 2023, and December 1, 2024, were included as study subjects. Serum and clinical data were collected prospectively. Infants diagnosed with NEC at Bell stage II or above were included in the NEC group. For the control group, preterm infants hospitalized during the same period were selected based on matching criteria of GA ± 3 days and birth weight ± 100 g, following a 1:1 randomization principle.

The study was approved by the Ethics Committee of Children’s Hospital of Soochow University and Suzhou Hospital of Nanjing Medical University (No. 2023CS130; approval date: January 12, 2023). It conformed to the Declaration of Helsinki 1964 and its subsequent amendments or comparable ethical standards. The study obtained informed consent from the guardians of the children and agreed to the use and disclosure of their clinical data and full written authorization were obtained.

### 2.2 Exclusion criteria

(1) Definite presence of inherited metabolic and chromosomal disorders. (2) Presence of severe congenital structural malformations. (3) The clinical data is incomplete, mainly due to the lack of key imaging examinations (such as abdominal X-ray/ultrasound) and patients being discharged automatically due to a short hospital stay, making it impossible to identify whether they developed NEC.(4) Refusal of the child’s guardian to participate in the study.

### 2.3 Biological sample collection and serum DUOX2 testing

To ensure the scientific accuracy of the detection time and the reliability of sample sources, this study relies on the preterm infant cohort database and biobank established by the *Suzhou Municipal Key Laboratory of Neonatal Major Disease Rescue and Early-Life Prevention of Adult Chronic Diseases*. As part of routine clinical procedures, residual serum samples from preterm infants were collected after routine laboratory tests (at 24 hours, 3 days, 1 week, and weekly thereafter). These samples were included in the biobank with informed consent from the guardians. All samples were processed within 2 hours of collection, centrifuged, aliquoted, and stored at -80°C in a frozen storage facility. Samples were assigned a unique identification number and underwent quality control to prevent bias due to inconsistent sample handling. The “index time” for NEC cases was defined as the clinical diagnosis time.

Serum samples collected within 3–5 days prior to the clinical diagnosis of NEC were selected for DUOX2 measurement. The control group serum samples were collected at time points matched to those of the NEC group, with an allowable deviation of ±3 days. Serum DUOX2 was detected by Human DUOX2 ELISA Kit (CAS: ELK3846, ELK Biotechnology, China), and the procedure was carried out according to the instruction of the kit.

### 2.4 Diagnostic criteria and definitions

NEC diagnosis and staging based on modified Bell staging criteria (Kim and Seo, 2020; Roberts et al., 2024). All neonates meeting NEC stage II or above were included in the NEC group. Small for gestational age (SGA), Respiratory Distress Syndrome (RDS), hemodynamically significant Patent Ductus Arteriosus (hsPDA), atrial septal defect (ASD), ventricular septal defect (VSD), early-onset sepsis (EOS) and late-onset sepsis (LOS) diagnosis reference *Practical Neonatology* (Shao et al., 2019).

### 2.5 Clinical data collection

Clinical data collection primarily included baseline characteristics of the infants, such as sex, gestational age (GA), birth weight (BW), mode of delivery, use of assisted reproductive technology (ART), and duration of premature rupture of membranes (PROM). Maternal data included age, underlying conditions, and comorbidities during pregnancy—specifically gestational hypertension (GH), gestational diabetes mellitus (GDM), and chorioamnionitis—as well as prenatal exposure to antibiotics, glucocorticoids, and magnesium sulfate.

In addition, we collected information on the infants’ primary treatments, laboratory tests, and imaging examinations during hospitalization. Primary treatments included oxygen therapy, antibiotics, glucocorticoids, and red blood cell transfusions. Laboratory data were obtained from routine blood and biochemical tests performed around the time of serum DUOX2 measurement. Imaging examinations primarily included frontal and lateral abdominal radiographs and abdominal ultrasound. All clinical data, laboratory results, and records of underlying diseases or comorbidities were collected prior to the onset of NEC, and no suspected NEC-related symptoms were present at the time of data collection.

### 2.6 Bioinformatics analysis

The GSE64801 dataset used in this study consists of publicly available high-throughput sequencing data, obtained from the GEO database of the National Center for Biotechnology Information (NCBI). According to the original study (Tremblay et al., 2016), sequencing samples were collected from ileal tissues of infants diagnosed with Bell stage III NEC during surgical resection (n=9), while control samples (n=5) were obtained from the ileal tissues of non-NEC neonates undergoing surgical treatment. Differential gene expression analysis was conducted using the DESeq2 package. Gene set enrichment analysis was performed in R (v4.2.1) using the clusterProfiler package. Receiver operating characteristic (ROC) curves were generated using the pROC package (v1.18.0) to assess the diagnostic performance of standardized gene expression data, with the area under the curve (AUC) and 95% confidence intervals calculated based on 1,000 bootstrap replicates. The optimal cutoff point was determined using Youden’s index. For immune infiltration analysis, the expression of DUOX2 was correlated with the relative abundance of 22 immune cell types (estimated using CIBERSORT) in case samples, employing Spearman’s rank correlation test (*P* < 0.05). Significant associations were visualized using annotated scatterplots. All graphs were plotted using ggplot2.

### 2.7 Statistical analysis

Data were statistically analyzed using R (4.2.1) and GraphPad Prism 9 software. Normally distributed continuous variables are presented as the mean ± standard deviation, and comparisons between the two groups were performed using the t-test. Non-normally distributed continuous variables are presented as the median (25th percentile, 75th percentile) [M(P25, P75)], and comparisons between the two groups were conducted using the Mann-Whitney U test. The chi-square test or Fisher’s exact probability method was used to analyze the categorical variables. *Spearman* correlation analysis was used to assess the relationship between serum DUOX2 levels and other baseline laboratory indicators, with the analysis conducted across the entire enrolled population. Multiple linear regression analysis was conducted on DUOX2 and laboratory differential indicators, controlling for GA, BW, and CRP. Univariate analysis variables with statistical differences were analyzed by multivariate logistic regression analysis. ROC curves of serum DUOX2 for NEC prediction were plotted. *p* < 0.05 was considered statistically significant.

## 3 Results

### 3.1 DUOX2 is significantly upregulated in intestinal tissues of NEC patients

**Figure 1:**
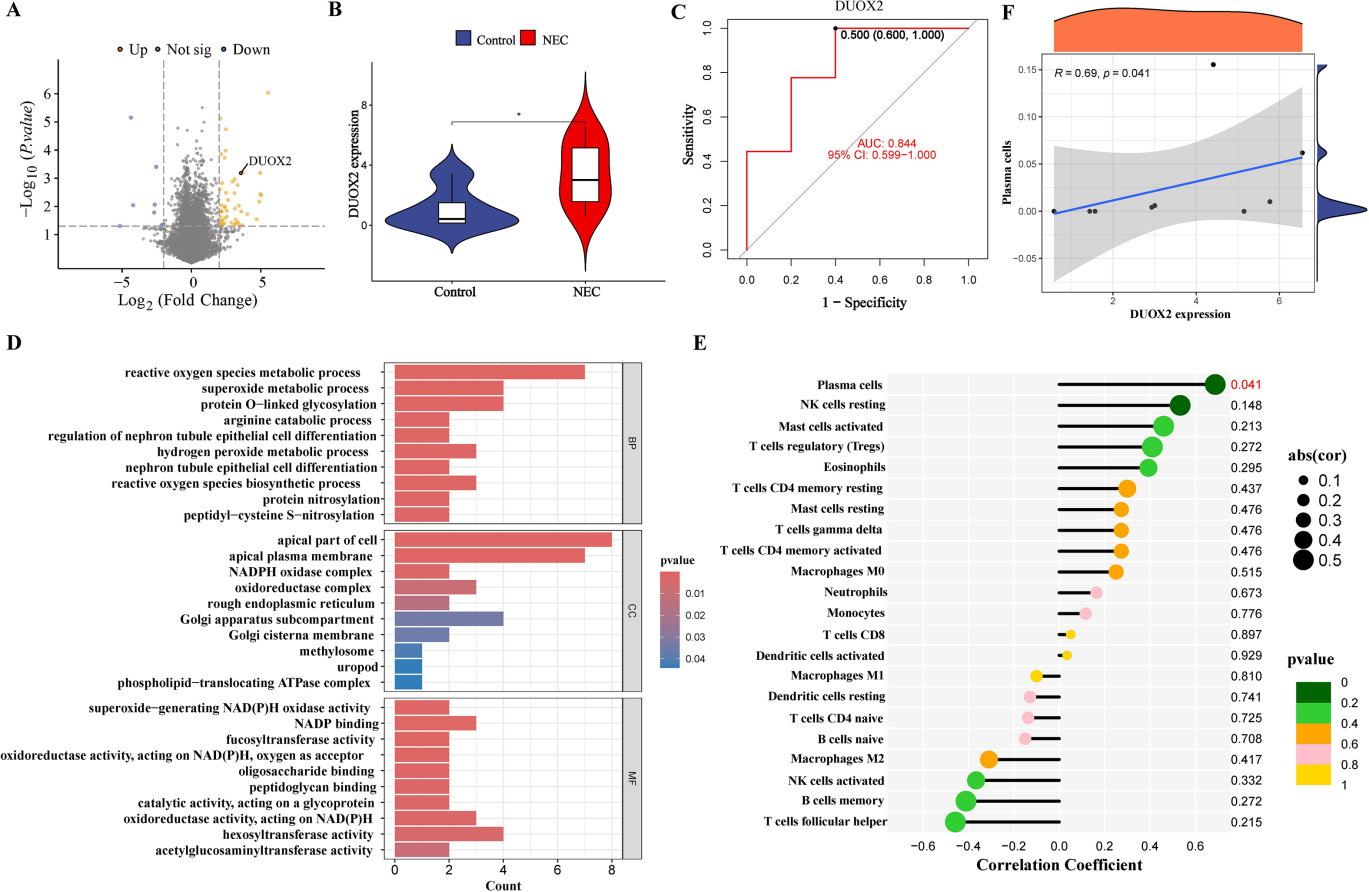
GEO data of intestinal tissue from NEC patients (GSE64801) were analyzed. Differential gene analysis of the NEC dataset (GSE64801) identified 71 differentially expressed genes (DEGs) between the NEC (n=9) and Control (n=5) groups. Of these, 18 genes were upregulated (yellow dot cluster) and 53 were downregulated (purple dot cluster) in NEC intestinal tissue. Notably, DUOX2 was significantly overexpressed in the intestinal tissue of NEC patients (*P*<0.05) **(A, B)**, with an AUC value of 0.844 (0.599-1.000) predicting the occurrence of NEC **(C)**.The GO enrichment analysis revealed that the DEGs between the two groups mainly affect redox reactions, including reactive oxygen species (ROS) and superoxide **(D)**. Immunoinfiltration analysis revealed a positive correlation between DUOX2 expression and plasma cells (R=0.69, *P*=0.041) **(E, F)**. NEC, Necrotizing Enterocolitis; DUOX2, dual oxidase 2. *P<0.05.

Differential gene identification was performed on the NEC dataset (GSE64801), resulting in the identification of 71 differentially expressed genes (DEGs) between the two groups. Among these, 18 genes were significantly upregulated in NEC intestinal tissue (yellow dot cluster), while 53 genes were downregulated (purple dot cluster) (**Figure 1A**), in which the top three protein-coding genes that were significantly upregulated in NEC intestinal tissues were CD24, DUOX2, and LYPD8. DUOX2, which has not yet been intensively studied and explored in the NEC, has been shown to be strongly associated with the gut innate defense response, with abnormalities in gut microbes and with an increased risk of inflammation (El et al., 2005; Grasberger et al., 2021). Further analysis revealed that DUOX2 was significantly highly expressed in NEC intestinal tissues (*P*<0.05) (**Figure 1B**), with an AUC value of 0.844 (0.599-1.000) predicting the occurrence of NEC (**Figure 1C**). The GO enrichment analysis revealed that the DEGs between the two groups mainly affect redox reactions, including ROS and superoxide (**Figure 1D**), which are similar to the biological activity of DUOX2. Immunoinfiltration analysis revealed a positive correlation between DUOX2 expression and plasma cells (R=0.69, *P*=0.041) (**Figures 1E, F**).

### 3.2 Clinical characteristics of NEC patients

**Figure 2:**
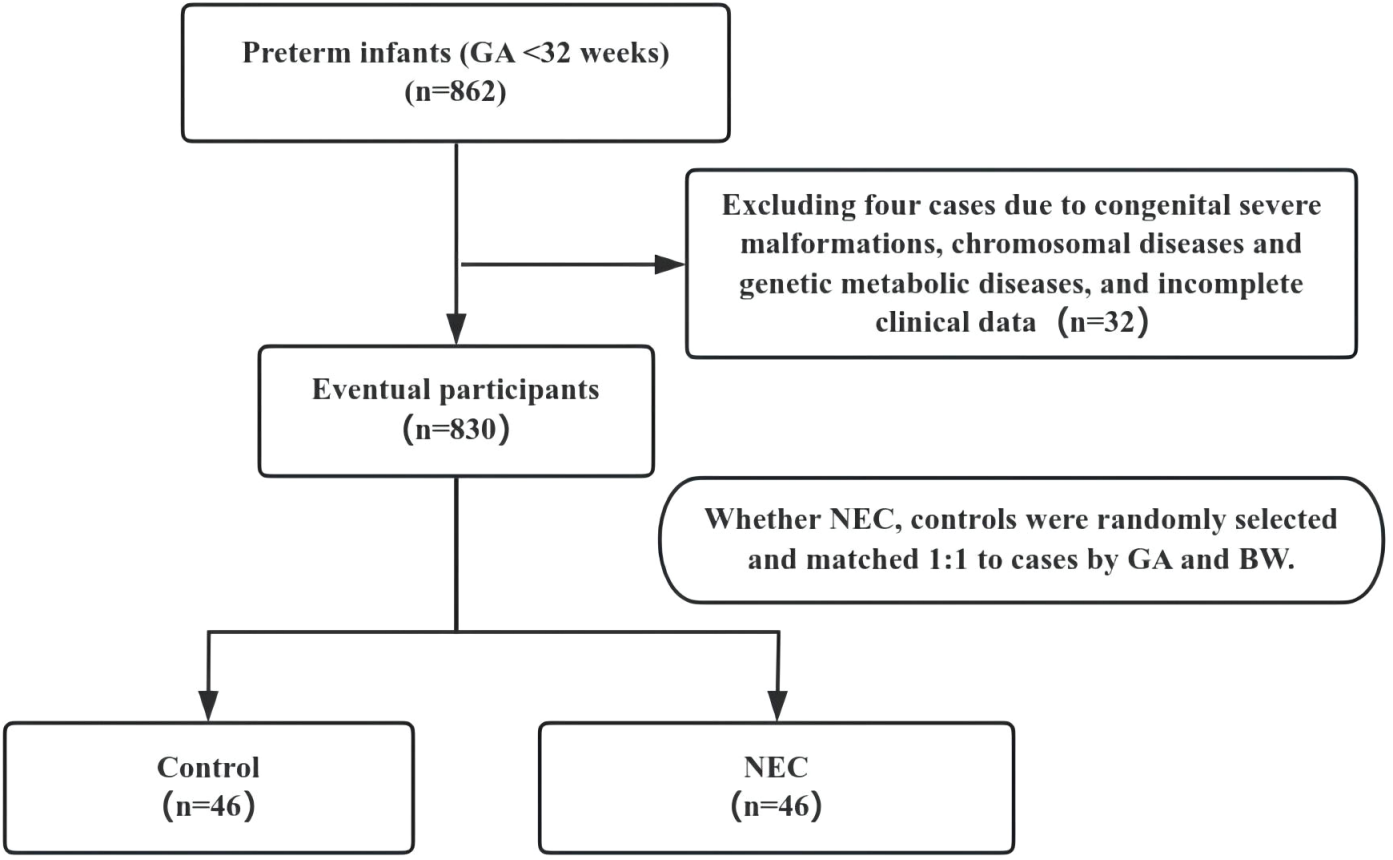
The study enrollment flow diagram. GA, gestational age; BW, birth weight; NEC, Necrotizing Enterocolitis.

The study enrollment flow diagram, including detailed reasons for exclusion, is shown in **Figure 2**. A total of 862 preterm infants with gestational age <32 weeks were initially eligible. Among them, 32 infants were excluded: 8 due to major congenital malformations, 9 with confirmed chromosomal abnormalities or genetic metabolic disorders, and 15 with incomplete clinical data. After applying these criteria, 46 infants (5.34%) with NEC (Bell stage≥II) met the enrollment requirements. Of these, 33 were classified as stage II and 13 as stage III, with 17 infants undergoing surgical procedures.

The study enrollment flow diagram, including reasons for exclusion, is presented in **Figure 2**. There were 862 cases of preterm infants with gestational age <32 weeks. 32 cases of congenital severe malformations, chromosomal diseases and genetic metabolic diseases, and incomplete clinical data were excluded, and a total of 46 cases finally met the criteria for enrollment in the NEC, of which 33 cases were in Stage II, 13 cases were in Stage III, and 17 cases were undergoing surgical procedures. The mean GA of the children in the NEC group was 29.50 (28.11, 30.54) weeks, mean BW was 1175.00 (997.50, 1357.50) g, and mean day of onset of NEC was (23 ± 12) days. Forty-six infants who met the criteria for enrollment were randomly selected based on gestational age and birth weight as the control group.

Demographic characteristics, clinical characteristics, and primary treatment during hospitalization for the NEC and control groups are shown in **Table 1**. There was no significant difference between the two groups of children in terms of maternal age, ART, GH, GDM, PROM>18h, prenatal glucocorticoids, prenatal MgSO4, prenatal antibiotics, cesarean section, gender, GA, BW, SGA, RDs, ASD, VSD, EOS, antibiotic, glucocorticoid, mechanical ventilation, days of mechanical ventilation, and RBC transfusions (*P*>0.05). Compared to the control group, hsPDA, LOS, Days of antibiotic and number of RBC transfusions ≥3 were significantly higher in the NEC group (*P*<0.05).

**Table 1:** Clinical characteristics, underlying diseases, and main treatments of the study cohort.

Characteristics	NEC (n=46)	Non-NEC (n=46)	*P*
Maternal characteristics			
Maternal age, years, Median (P25,P75)	30 (32, 27)	30 (27, 34)	0.397
Assisted reproductive technology (n,%)	6 (13.04)	7 (15.22)	0.765
Gestational hypertension (n,%)	15 (32.61)	9 (19.57)	0.154
Gestational diabetes mellitus (n,%)	12 (26.09)	15 (32.61)	0.492
PROM>18h (n,%)	11 (23.91)	6 (13.04)	0.179
Chorioamnionitis (n,%)	1 (2.17)	1 (2.17)	1.000
Antenatal antibiotics (n,%)	13 (28.26)	13 (28.26)	1.000
Antenatal MgSO4 (n,%)	21 (45.65)	18 (39.13)	0.527
Antenatal Steroids (n,%)	35 (76.08)	36 (78.26)	0.804
Caesarean Section (n,%)	28 (60.87)	25 (54.35)	0.527
Infant characteristics			
Female (n,%)	21 (45.65)	20 (43.48)	0.834
GA, weeks	29.50 (28.11, 30.54)	29.64 (28.57, 31.00)	0.312
Birth weight, g	1175.00 (997.50, 1357.50)	1150.00 (950.00, 1312.50)	0.407
Small for GA (n,%)	18 (39.13)	13 (28.26)	0.270
Underlying diseases and comorbidities			
Respiratory distress syndrome (n,%)	29 (63.04)	28 (60.87)	0.601
hsPDA (n,%)	24 (52.17)	13 (28.26)	0.019
Atrial septal defect (n,%)	25 (54.35)	22 (47.83)	0.532
Ventricular septal defects (n,%)	2 (4.35)	1 (2.17)	1.000
Early-Onset Sepsis (n,%)	1	0	1.000
Late-Onset Sepsis (n,%)	13 (28.89)	5 (10.87)	0.036
Main treatment			
Antibiotic (n,%)	46 (100.00)	43 (93.48)	0.078
Days of antibiotic, days	24.50 (12.75, 32.00)	13.00 (7.75, 20.25)	0.001
Mechanical ventilation (n,%)	13 (28.26)	10 (21.74)	0.470
Days of mechanical ventilation, days	0 (0, 5)	0 (0, 2)	0.463
RBC transfusions (n,%)	33 (71.74)	29 (63.04)	0.374
Number of RBC transfusions ≥3 (n,%)	23 (50.00)	12 (26.09)	0.018
Glucocorticoid (n,%)	4 (8.70)	7 (15.22)	0.335

NEC, Necrotizing Enterocolitis; PROM, Premature Rupture of Membranes; GA, gestational age; hsPDA, hemodynamically significant Patent Ductus Arteriosus; RBC, red blood cell.

### 3.3 Laboratory tests

Laboratory investigations in the two groups are shown in **Table 2**. There was no significant difference between the two groups in the levels of white blood cell count, platelet count, platelet distribution width, mean platelet volume, plateletcrit, hemoglobin, mean corpuscular volume, hematocrit, RBC distribution width (RDW), C-reactive protein, albumin, total bilirubin, alkaline phosphatase, lactic acid, and glucocholic acid (*P*>0.05). Lactate dehydrogenase levels were significantly increased in children in the NEC group compared to the control group (*P*<0.05).

**Table 2:** Laboratory tests in the study cohort.

Characteristics	NEC (n=46)	Non-NEC (n=46)	*P*
White blood cell counts, ×10^9^/L	10.50 ± 5.44	11.26 ± 4.29	0.461
Platelet counts, ×10^9^/L	218.00 (145.50, 449.50)	341.50 (241.75, 397.75)	0.129
Platelet distribution width, %	13.56 ± 2.73	13.96 ± 3.24	0.528
Mean platelet volume, fL	11.16 ± 1.11	11.43 ± 1.10	0.251
Plateletcrit, %	0.33 ± 0.15	0.36 ± 0.13	0.323
Hemoglobin, g/L	112.89 ± 23.46	121.80 ± 20.99	0.060
Mean corpuscular volume, fL	97.08 ± 7.19	99.90 ± 7.25	0.065
Hematocrit, L/L	0.34 ± 0.72	0.36 ± 0.64	0.186
Red blood cell distribution width, %	16.68 ± 2.46	15.92 ± 1.39	0.074
C-reactive protein, mg/L	16.35 (1.37, 41.55)	12.59 (4.33, 15.21)	0.842
Albumin, mg/L	31.18 ± 3.08	32.63 ± 3.73	0.075
Total bilirubin, umol/L	93.87 ± 58.50	85.49 ± 55.33	0.548
Alkaline phosphatase, U/L	260.18 ± 145.18	260.90 ± 133.85	0.982
Lactate dehydrogenase, U/L	407.43 ± 141.95	344.47 ± 109.63	0.029
Lactic acid, U/L	3.34 ± 2.33	2.66 ± 1.35	0.207
Glucocholic acid, U/L	12.57 ± 12.16	10.16 ± 6.34	0.277

NEC, Necrotizing Enterocolitis.

### 3.4 Serum DUOX2 levels and their correlation with other laboratory parameters

**Figure 3:**
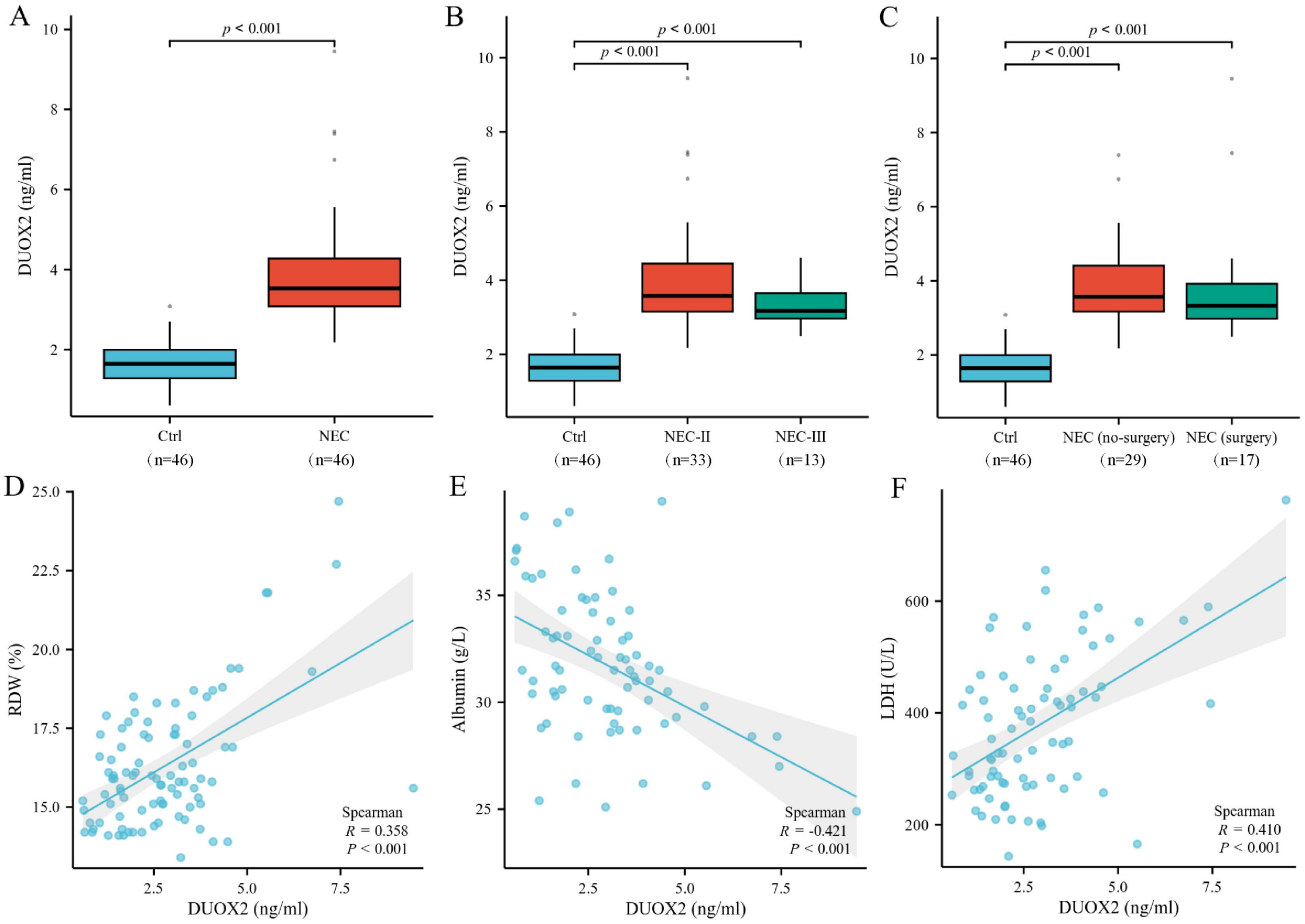
Serum DUOX2 levels and their correlation with other laboratory parameters. The serum DUOX2 levels of children with NEC in the NEC group (n=46), NEC stage II (n=33), NEC stage III (n=13), conservative medical treatment group (n=29), and surgical group (n=17) were significantly higher than those in the control group (n=46) (*P*<0.05) **(A-C)**. Serum DUOX2 levels showed a significant positive correlation with RDW (R=0.358, *P*<0.05) and LDH (R=0.410, *P*<0.05), a significant negative correlation with albumin levels (R=-0.420, *P*<0.05) **(D-F)**. DUOX2, dual oxidase 2; NEC, Necrotizing Enterocolitis; RDW, red cell distribution width; LDH, lactate dehydrogenase. The Spearman correlation analysis was performed on the entire enrolled population.

Serum DUOX2 levels of different subgroups are shown in **Figures 3A–C**. The GA and BW of NEC III degree children were 29.00 (28.29, 30.14) weeks and 1150.00 (1000.00, 1345.00) g, respectively; for NEC II degree neonates, 29.57 (28.00, 30.93) weeks and 1250.00 (980.00, 1375.00) g; for the surgical treatment group, 29.29 (28.21, 30.21) weeks and 1250.00 (985.00, 1375.00) g; and for the conservative medical treatment group, 29.57 (28.57, 31.00) weeks and 1150.00 (1000.00, 1300.00) g. No significant differences in GA and BW were found between the subgroups (*P*>0.05). The serum DUOX2 levels of children with NEC in the NEC group, NEC stage II, NEC stage III, conservative medical treatment group, and surgical group were significantly higher than those in the control group (*P*<0.05). There was no significant difference in serum DUOX2 levels between subgroups of NEC (*P*>0.05). Correlation analysis of serum DUOX2 and other laboratory parameters is shown in **Figures 3D–F**. Serum DUOX2 levels showed a significant positive correlation with RDW (R=0.358, *P*<0.05) and LDH (R=0.410, *P*<0.05), a significant negative correlation with albumin levels (R=-0.420, *P*<0.05), and no significant correlation with other laboratory parameters.

Further multiple linear regression analysis, controlling for gestational age, birth weight, and CRP, showed that serum DUOX2 levels remained independently positively correlated with RDW (β=0.86, [95% CI 0.03–1.69], *P* = 0.043) and LDH (β=74.7, [95% CI 33.9–115.4], *P* = 0.002), while showing a negative trend with albumin (β= -0.93, [95% CI -2.11–0.26], *P* = 0.113), though this did not reach statistical significance.

### 3.5 Analysis of risk factors for NEC in preterm infants with GA <32 weeks

As shown in **Table 3**, multifactorial analysis using logistic regression analysis of risk factors with significant differences in univariate analysis revealed that hsPDA, days of antibiotic, number of RBC transfusions ≥3, and serum DUOX2 level were risk factors for NEC in preterm infants with GA <32 weeks.

**Table 3:** Analysis of risk factors for NEC in preterm infants with GA <32 weeks.

Characteristics	OR	95%CI	*P-value*
hsPDA	18.890	2.827 – 126.223	0.002
LOS	4.277	0.521 – 35.149	0.176
Days of antibiotic	1.139	1.052 – 1.234	0.001
Number of RBC transfusions ≥3	1.662	1.095 – 2.522	0.017
LDH	0.996	0.990 – 1.001	0.143
DUOX2	3.526	1.690 – 7.355	0.000

NEC, Necrotizing Enterocolitis; GA, gestational age; hsPDA, hemodynamically significant Patent Ductus Arteriosus; RBC, red blood cell; LDH, lactate dehydrogenase; DUOX2, dual oxidase 2.

### 3.6 ROC curves for DUOX2 on the presence of NEC

**Figure 4:**
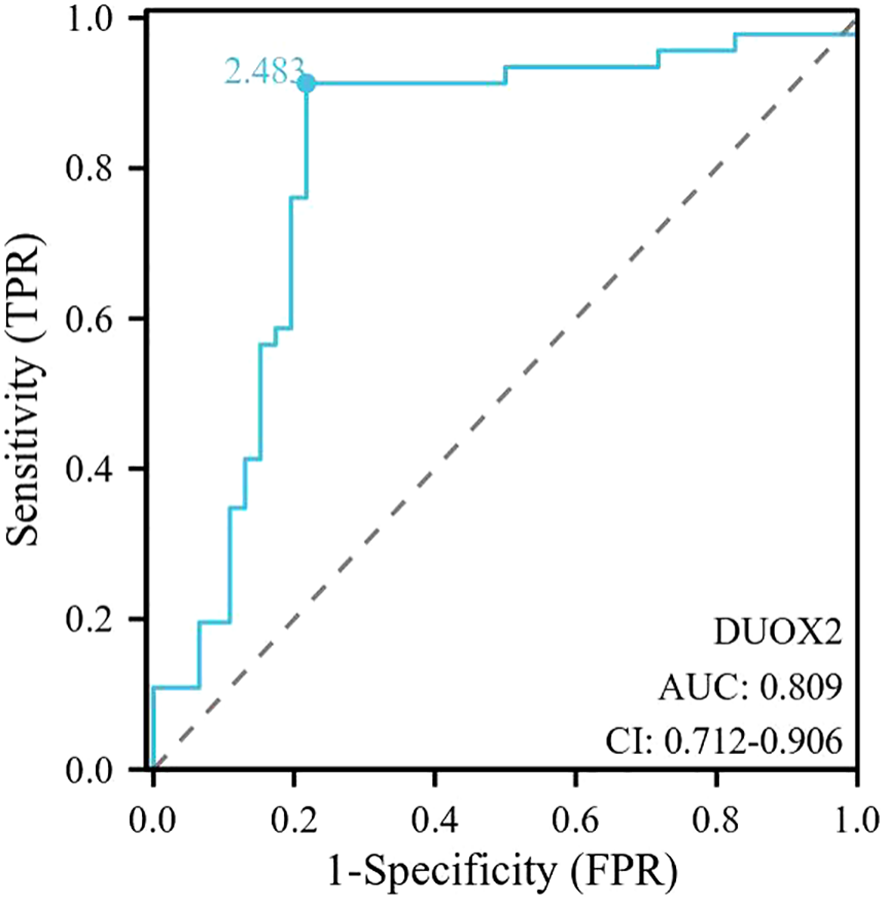
ROC curves for DUOX2 on the presence of NEC. The AUC value of serum DUOX2 level for NEC in preterm infants with GA <32 weeks was 0.809 (0.712- 0.906), with an optimal cut-off value of 2.483, a sensitivity of 91.30% and a specificity of 78.26%, positive predictive value was 80.44%, and negative predictive value was 86.84%. ROC, receiver operating characteristic; DUOX2, dual oxidase 2; NEC, Necrotizing Enterocolitis; GA, gestational age.

As shown in **Figure 4**, the AUC value of serum DUOX2 level for NEC in preterm infants with GA <32 weeks was 0.809 (0.712- 0.906), with an optimal cut-off value of 2.483, the sensitivity was 91.30%, specificity was 78.26%, positive predictive value was 80.44%, and negative predictive value was 86.84%.

## 4 Discussion

The occurrence of NEC is primarily driven by oxidative stress, abnormal microbial colonization, dysregulated metabolic pathways, inappropriate feeding, infection, inflammatory responses, and other factors acting on the immature intestinal barrier of preterm infants (Qian et al., 2017; Mihi et al., 2021; Gershner and Hunter, 2024; Panigrahi, 2024; Subramanian et al., 2024; Wang et al., 2024). We conducted GO enrichment analysis on the differentially expressed genes in the intestinal tissue of NEC neonates and similarly found that oxidative stress plays an important role in the development of NEC. Therefore, from the perspective of oxidative stress, identifying novel biomarkers and disease targets is a crucial breakthrough for the prevention and treatment of NEC.

DUOX is a NADPH oxidase located on the cell membrane that participates in various physiological and pathological processes. DUOX, expressed on the apical membrane of intestinal epithelial cells in metazoans, is involved in the release of H_2_O_2_, one of the most primitive innate defense mechanisms in the gut (Ha et al., 2005). DUOX exists in two isoforms, DUOX1 and DUOX2, with the former enriched in the bronchial and uroepithelial tissues, and the latter predominantly expressed in the intestine (Geiszt et al., 2003; El et al., 2005). With continued research and deeper understanding of DUOX2, numerous studies have identified it as a key host factor in maintaining gut microbial and immune homeostasis, playing a critical role in mammalian immune regulation and dysbiosis-related diseases (Csillag et al., 2007; Grasberger et al., 2015; Grasberger et al., 2021). Grasberger et al. (2021)) showed that the deleterious DUOX2 variant is associated with elevated plasma levels of interleukin 17C and an increase in specific Aspergillus phylum pathogens, which are associated with an increased risk of inflammatory bowel disease. Recent studies have shown that elevated DUOX2 contributes to intestinal epithelial barrier dysfunction, microbiome alterations, and subclinical inflammation in IBD, while inhibition of its activation significantly improves disease outcomes (Hazime et al., 2025).

In our study, immune infiltration analysis showed a positive correlation between DUOX2 expression and plasma cell abundance. Although this correlation does not establish a direct cause-and-effect relationship, it suggests that oxidative stress associated with DUOX2 might create a gut microenvironment conducive to plasma cell infiltration and activation. Previous studies have demonstrated that DUOX2 modulates epithelial barrier integrity and local redox signaling through the production of hydrogen peroxide (Grasberger et al., 2015), while plasma cells play a crucial role in mucosal immunity by amplifying intestinal inflammation and disrupting the epithelial barrier (Wang et al., 2019). Therefore, we hypothesize that the high expression of DUOX2, through enhanced oxidative stress and epithelial stress responses, may indirectly promote the recruitment or activation of plasma cells at NEC lesions. Furthermore, plasma cell infiltration and proliferation can impair the matrix-epithelial regeneration signaling required for mucosal healing, exacerbating intestinal inflammation (Frede et al., 2022). Although the current correlation analysis does not establish causality, these findings suggest a potential biological interaction between epithelial oxidative signaling and immune cell infiltration, which may contribute to the pathogenesis of NEC.

Although DUOX2 has been shown to play an important role in intestinal inflammation as well as microbial colonization and invasion, no study to date has reported a correlation between DUOX2 and the occurrence of NEC. In the present study, analysis of the GEO database revealed significantly elevated DUOX2 expression in intestinal tissues of children with NEC. Clinical testing of serum samples further demonstrated that serum DUOX2 levels were significantly higher in children with NEC, across varying severities and in surgically treated cases, compared with the control group. Moreover, elevated serum DUOX2 was identified as an independent risk factor for NEC development. However, no significant differences in serum DUOX2 levels were observed among the different NEC subgroups, which may be attributable to the limited sample size in our study.

We performed a correlation analysis between serum DUOX2 and other laboratory variables in the study population and found a negative correlation between serum DUOX2 and albumin levels. Albumin serves multiple physiological functions, including scavenging free radicals, mitigating oxidative stress– and inflammation-induced cellular damage, and maintaining immune cell homeostasis, all of which influence disease onset and prognosis (Soeters et al., 2019; Hao et al., 2024). In NEC, systemic inflammation and barrier dysfunction may underlie the coexistence of elevated DUOX2 expression and hypoalbuminemia. While albumin decline likely reflects protein-losing enteropathy and enhanced catabolism, DUOX2 upregulation may exacerbate mucosal damage and inflammation, reinforcing a vicious cycle. In addition, we found that serum DUOX2 levels were significantly and positively correlated with RDW and LDH. RDW is mainly used to assess the size heterogeneity of red blood cells. Studies have shown that host inflammatory responses and oxidative stress affect erythropoiesis, as well as red blood cell morphology and function, ultimately leading to elevated red cell distribution width (RDW), which is closely associated with the severity and prognosis of inflammatory diseases (Hao et al., 2024; Xiong et al., 2024; Xu et al., 2024). LDH has long been shown to strongly correlate with intestinal inflammatory responses and barrier damage, and positively correlates with inflammatory mediators such as interleukin-1β (IL-1β) (Fu et al., 2022; Li et al., 2024; Banguera-Ordonez et al., 2025). In addition, it has been shown that LDH levels may interact with the gut microbiota (Huang et al., 2023; Barron et al., 2024). This suggests that DUOX2 interacts with LDH and RDW in NEC preterm infants, but the specific regulatory mechanisms need to be further elucidated.

However, our study has several limitations. DUOX2 levels may be influenced by the postnatal age at which serum samples are collected; since DUOX2 also participates in broader inflammatory responses, its reliability as a definitive biomarker specific to NEC requires further validation in cohorts presenting with non-NEC intestinal inflammation. As DUOX2 is also involved in general inflammatory responses, its utility as a definitive NEC-specific biomarker requires further validation in cohorts with non-NEC intestinal inflammation. Although this study demonstrates the upregulation of DUOX2 in NEC and its association with plasma cells and redox reactions, clinical biomarker measurements do not yet reveal the specific mechanisms by which DUOX2 contributes to the development of NEC. Further research, such as DUOX2 inhibition or knockout models, is needed to clarify its role in the pathogenesis of NEC. Furthermore, due to bioeconomic constraints, we were unable to perform serial measurements of serum DUOX2 levels to assess their dynamic changes during NEC progression, which is essential for determining optimal biomarker sampling timepoints. Finally, the relatively small number of preterm infants included in this study may explain the lack of significant differences in serum DUOX2 levels among NEC subgroups. Larger, more diverse cohorts are needed in future studies to confirm the clinical value of DUOX2 in NEC.

## 5 Conclusion

Our study demonstrated that DUOX2 levels were significantly elevated in NEC patients and independently associated with disease severity, showing correlations with RDW, LDH, and hypoalbuminemia. These findings suggest that elevated DUOX2 levels are associated with the development of NEC in preterm infants.

## Data Availability

The original contributions presented in the study are included in the article/supplementary material. Further inquiries can be directed to the corresponding author/s.

## Glossary

ART: assisted reproductive technology
ASD: atrial septal defect
BW: birth weight
DUOX2: Dual Oxidase 2
ELISA: Enzyme-linked Immunosorbent Assay
GA: gestational age
GDM: gestational diabetes mellitus
GH: gestational hypertension
hsPDA: hemodynamically significant patent ductus arteriosus
LDH: lactate dehydrogenase
NEC: neonatal necrotizing enterocolitis
PROM: premature rupture of membranes
ROS: reactive oxygen species
RDs: respiratory distress syndrome
RDW: red blood cell distribution width
SGA: small for gestational age
VSD: ventricular septal defect
